# Prevalence and Predictors of Anxiety and Depression Among Teachers in Newfoundland and Labrador, Alberta, and Nova Scotia

**DOI:** 10.1192/j.eurpsy.2026.11447

**Published:** 2026-07-31

**Authors:** B. Agyapong, Y. Wei, V. I. O. Agyapong

**Affiliations:** 1 Psychiatry, University of Alberta, Edmonton; 2 Psychiatry, Dalhousie University, Halifax, Canada

## Abstract

**Introduction:**

Globally, anxiety and depression are leading causes of work-related disability and have profound effects on educators’ mental and physical well-being. These disorders contribute to reduced productivity, significant personal distress, and substantial economic and public health burdens.

**Objectives:**

To determine the prevalence and independent predictors of likely Generalized Anxiety Disorder (GAD) and likely Major Depressive Disorder (MDD) among teachers in Newfoundland and Labrador, Alberta, and Nova Scotia.

**Methods:**

This cross-sectional study surveyed educators enrolled in the *Wellness4Teachers* program, a daily supportive text messaging initiative. Participants completed an online questionnaire assessing likely GAD and MDD using the Generalized Anxiety Disorder-7 (GAD-7) and Patient Health Questionnaire-9 (PHQ-9) scales. Data were analyzed using SPSS version 28 (IBM Corp).

**Results:**

Of 1,912 *Wellness4Teachers* subscribers, 763 completed the survey (response rate: 39.9%). The prevalence of likely Major Depressive Disorder (MDD) and likely Generalized Anxiety Disorder (GAD) was 55.7% and 46.0%, respectively. After adjusting for all other variables in the regression model, participants reporting high stress were significantly more likely to experience both MDD (OR = 7.24, 95% CI: 4.22-12.42) and GAD (OR = 7.40, 95% CI: 4.63-11.80) compared to those with mild to moderate stress. Teachers experiencing emotional exhaustion were also more likely to report MDD (OR = 4.92, 95% CI: 3.01-8.05) and GAD (OR = 4.34, 95% CI: 2.47-7.62). Those with a low sense of professional accomplishment were 2.13 times more likely to exhibit MDD symptoms (OR = 2.13, 95% CI: 1.41-3.23) and 1.52 times more likely to report GAD symptoms (OR = 1.52, 95% CI: 1.01-2.29). Similarly, low resilience was associated with higher odds of both likely MDD (OR = 1.82, 95% CI: 1.24-2.66) and likely GAD (OR = 3.01, 95% CI: 2.03-7.62) compared to normal or high resilience. Teachers with more than 20 years of experience were less likely to experience GAD symptoms than those with five years or fewer (OR = 0.28, 95% CI: 0.12-0.64). No other sociodemographic or work-related factors independently predicted likely MDD or GAD.

**Conclusions:**

Findings highlight high rates of anxiety and depression among Canadian teachers and emphasize the need for comprehensive mental health supports. Interventions that reduce stress and emotional exhaustion while strengthening resilience may help improve teacher well-being and educational outcomes.

**Disclosure of Interest:**

None Declared

